# Aptamer-Based Graphene Field-Effect Transistor Biosensor for Cytokine Detection in Undiluted Physiological Media for Cervical Carcinoma Diagnosis

**DOI:** 10.3390/bios15030138

**Published:** 2025-02-23

**Authors:** Ziran Wang, Wenting Dai, Zaiyu Zhang, Haipeng Wang

**Affiliations:** 1 Key Laboratory of High-Efficiency and Clean Mechanical Manufacture of MOE, School of Mechanical Engineering, Shandong University, Jinan 250100, China; 2 Department of Mechanical Engineering, Columbia University, New York, NY 10027, USA

**Keywords:** graphene field-effect transistor, biosensor, cytokine, physiological media, cervical carcinoma

## Abstract

Personalized monitoring of disease biomarkers is of great interest in women’s health. However, existing approaches typically involve invasive inspection or bulky equipment, making them challenging to implement at home. Hence, we present a general strategy for label-free and specific detection of disease biomarkers in physiological media using an aptamer-based biosensor. The biosensor is a graphene field-effect transistor that involves immobilizing the aptamer and a biomolecule-permeable polyethylene glycol (PEG) layer on the graphene surface. The aptamer is capable of specifically binding with the target biomarker, thus inducing a change in the sensing responses. The PEG layer can effectively reduce the nonspecific adsorption of nontarget molecules in the solution, and increase the effective Debye screening length in the region directly adjacent to the graphene. In this work, studies of a biosensor with modification of the aptamer and PEG show that cervical carcinoma biomarkers such as tumor necrosis factor-α and interleukin 6 can be sensitively and specifically detected in undiluted physiological media, with detection limits as low as 0.13 pM for TNF-a and 0.20 pM for IL-6. This work presents a significant method for the general application of the biosensor for disease diagnosis in women’s health.

## 1. Introduction

Cervical carcinoma, which gives rise to severe complications involving multiple systems such as the reproductive, digestive, and lymphatic systems, is a highly pathogenic malignant tumor for women. Recent studies indicate that cervical carcinoma primarily exhibits clinical symptoms such as abdominal discomfort and bloating, similar to other gynecological conditions [1,2]. Although imaging studies are useful in detecting abnormalities, the nonspecific symptoms of cervical carcinoma frequently make early diagnosis challenging. Consequently, it is highly desirable to develop a device for diagnosing and predicting the severity of ovarian cancer, offering the essential reference for the population’s self-diagnostics in daily life or aiding clinical diagnoses.

Physiological media (e.g., sweat and lavage fluid), containing numerous disease biomarkers, could potentially reflect the human body’s deeper physiological state [3,4,5]. Cytokines such as tumor necrosis factor alpha (TNF-α) and interleukin-6 (IL-6) have been reported in subjects with many severe diseases, such as cervical carcinoma and ovarian cancer [6,7]. Particularly, research shows that an abnormal concentration of cytokines in physiological media is critical in the diagnosis of cervical carcinoma or in predicting its severity [8,9]. Hence, developing biosensors that enable rapid and sensitive detection of cytokines in physiological media is of great significance for the diagnosing and monitoring of cervical carcinoma patients’ conditions [10,11,12].

Recently, aptamer-based biosensors have been developed to detect disease biomarkers. Such sensors involve using antibodies or aptamers as specific probes to recognize target molecules [13,14]. In particular, aptamer-based field-effect transistors configure from semiconductor materials [15], carbon nanotubes [16] and graphene [17] have received significant attention for biosensing applications. Among these materials, graphene is an ideal candidate as the conducting channel for biosensors, due to the unique characteristics such as high carrier mobility, low intrinsic noise and sensitive to its surface charge distribution [18,19,20]. Since the first investigation of biomarker detection using the graphene-based field-effect transistor (GFET) [21], such devices have demonstrated high sensitivity and specificity in label-free detection of various analytes, including ions, enzymes, small molecules, DNA, and proteins [22,23,24].

Biosensor development has been aimed at facilitating the creation of devices in the sensitive monitoring of biomarkers within authentic physiological media [25,26,27]. However, physiological media contains numerous biomolecules beyond the intended targets, encompassing metabolites (e.g., glucose and lactate), simple ions (e.g., Na^+^ and Ca^2+^) and cells. These nontarget molecules in such solutions tend to adsorb onto the graphene surface, introducing interference with biomarker detection. This interference reduces sensor sensitivity, making it challenging for the biosensor to effectively detect its targets in undiluted physiological media. Consequently, a necessity to investigate the capability of aptamer-based graphene biosensors arises, for sensitive detection of disease biomarkers in undiluted physiological media. To address nonspecific adsorption at graphene interfaces, reagents such as Nafion [28], Tween 20 [29], and, particularly, polyethylene glycol (PEG) [30] are utilized. PEG, recognized for its biocompatibility, enhances the Debye screening length in high ionic strength media, also offering its reliable attachment to device surfaces to reduce nonspecific interactions in physiological media [31,32]. However, these biosensors require FET analysis in PBS (phosphate-buffered saline) or diluted media, limiting their suitability for direct, in situ disease diagnostics under real clinical conditions. Consequently, there arises a need to investigate the capability of aptamer-based graphene biosensors for sensitive monitoring of disease biomarkers in physiological media.

**Figure 1:**
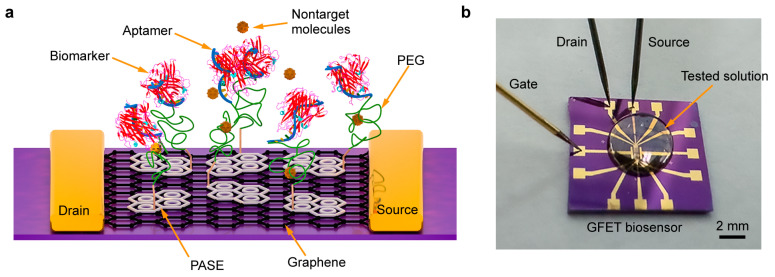
Aptamer-based GFET biosensor. (**a**) Schematic of the biosensor with the modification of PEG and aptamer used in this work. (**b**) Photograph of the biosensor for biomarker detection.

**Figure 2:**
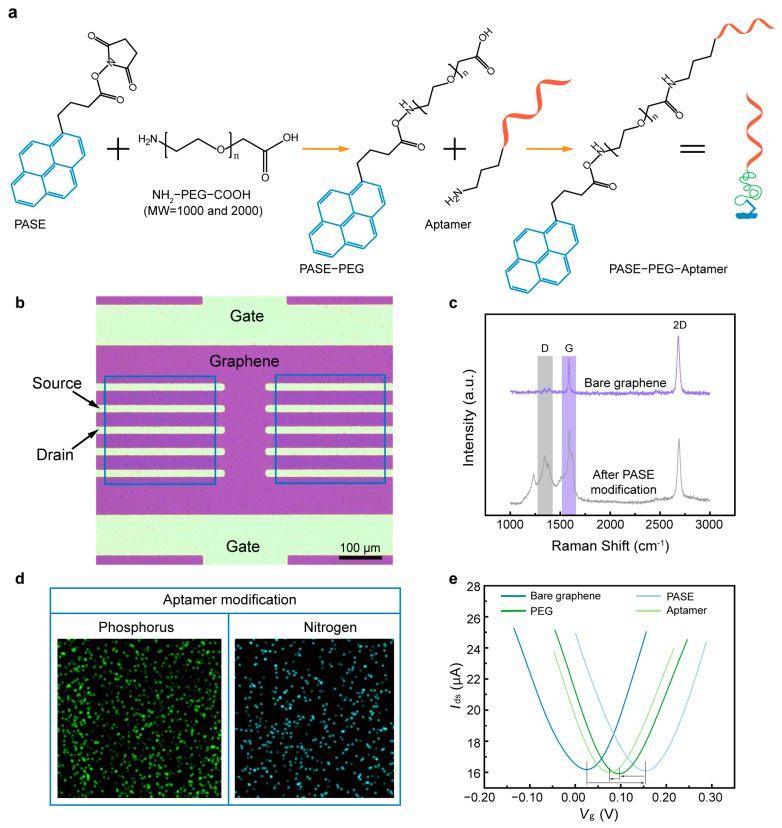
Surface modification of the GFET biosensor. (**a**) Schematic of the biosensor modification processes. (**b**) Microscope image of the biosensor with graphene conducting channel and electrodes. (**c**) Raman spectra of the graphene before and after PASE modification. (**d**) EDS of the biosensor after aptamer modification. (**e**) Transfer characteristic curves of functionalization processes of the graphene conducting channel.

This paper achieves a direct and sensitive measurement of cytokines in undiluted physiological media using an aptamer-based GFET biosensor modified with a biomolecule-permeable polyethylene glycol (PEG) isolation layer. The biosensor is configured from a graphene conducting channel covered with a PEG, and is functionalized with the aptamer specific to the biomarker to be measured (Figure 1a). Binding of the aptamer probe with the biomarker induces a change in the electric conductivity of the graphene, which is measured via the current between drain/source electrodes in solution environments to determine the cytokine concentration (Figure 1b). The modification of graphene with PEG isolation layer is investigated, which offers the effective elimination of interferences from the nonspecific adsorption and enhances the sensitivity of the biosensor (Figure 2a). Experimental results demonstrate that the biosensor enables sensitive detection of cytokines (e.g., TNF-α and IL-6), representative cervical carcinoma severity biomarkers, in undiluted physiological media (e.g., artificial sweat and lavage fluid) with limits of detection (LOD) as low as 0.13 pM for TNF-a and 0.20 pM for IL-6. With these capabilities, the aptamer-based GFET biosensor can be potentially used in daily noninvasive and rapid cervical carcinoma detection in human biofluids.

## 2. Materials and Methods

### 2.1. Materials

Chemical vapor deposition (CVD) graphene was purchased from Nanjing Muke Nanotechnology (Nanjing, China). 1-Pyrenebutyric acid N-hydroxysuccinimide ester (PASE), ethanolamine, 1-ethyl-3-(3-dimethylaminepropyl) carbodiimide hydrochloride (EDC•HCl), N-hydroxysulfosuccinimide (NHS) and human Interleukin-002 (IL-002) were ordered from Sigma-Aldrich. Various lengths of PEG polymers were purchased from Creative PEGWorks. Artificial sweat was purchased from Walgreens. TNF-α, IL-6 and IFN-gamma (IFN-γ) were purchased from R&D Systems. Aptamer (5′-NH_2_-TGG TGG ATG GCG CAG TCG GCG ACA A-3′) for TNF-α detection and 5′-NH_2_-GGT GGC AGG AGG ACT ATT TAT TTG CTT TTC T-3′ for IL-6 detection were synthesized and purified by Sangong Biotech (Shanghai, China).

### 2.2. Aptamer-Based Biosensor Design and Fabrication

The design and fabrication of the GFET biosensor is described in detail in the previous study [17]. Briefly, drain–source and on-chip gate electrodes were patterned onto SiO_2_ wafers using Ti/Au deposition (2 nm/38 nm), and then a monolayer graphene grown by chemical vapor deposition was transferred onto the drain–source electrode as a conducting channel. A microscope image of integrated GFET biosensor with graphene-based conducting channel (50 μm) is shown in Figure 2b. During operation, a sweeping gating voltage and consistent drain–source voltage were applied to the conducting channel, thereby generating drain–source current in the graphene. The change in the drain–source current arising from the aptamer–biomarker interaction is measured to determine the biomarker concentration.

### 2.3. Surface Modification and Characterization

The modification processes were carried out to enable cytokine detection by PEG and aptamer functionalization. 1-pyrenebutanoic acid succinimidyl ester (PASE) was first modified on the graphene through π-π stacking, which was used to immobilize NH_2_-PEG-COOH. Then, ethanolamine was used to quench the unreacted PASE. The mixture solution of EDC•HCl and NHS was used to activate the carboxylic group at the end of the PEG, enabling a condensation reaction of amino on the aptamer and activated carboxyl groups on the PEG. (Figure 2a) Finally, ethanolamine was used to occupy the unreacted PEG. The functionalization of graphene with PASE was verified using Raman spectra under 532 nm laser excitation (Figure 2c). The split of the G band and the remarkable D band were observed after PASE modification, confirming the coupling of graphene and pyrene groups on PASE. The successful modification of the aptamer was verified using energy dispersive spectroscopy (EDS). Phosphorus and nitrogen were observed to exist in significant amounts on the graphene surface; these are the unique constituent elements of the aptamer (Figure 2d). Further, the electrical signal of each modification process was investigated by analyzing the measured transfer characteristic curves (Figure 2e). The Dirac point (*V*_Dirac_) was observed to increase from 24 to 153 mV after PASE modification, and then decrease by 57 mV after NH_2_-PEG-COOH immobilization. Upon covalent coupling of the aptamer, *V*_Dirac_ decreased from 96 to 76 mV, suggesting that the modification induced n-type doping in the graphene. These results illustrate that the biosensor was successfully functionalized using PEG and aptamer.

## 3. Results

### 3.1. Detection of TNF-α in PBS

**Figure 3:**
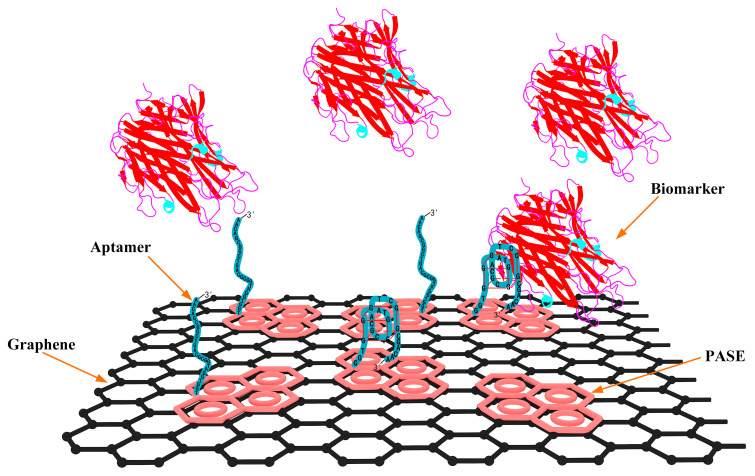
Schematic showing the conformation change in the aptamer upon binding with proteins.

**Figure 4:**
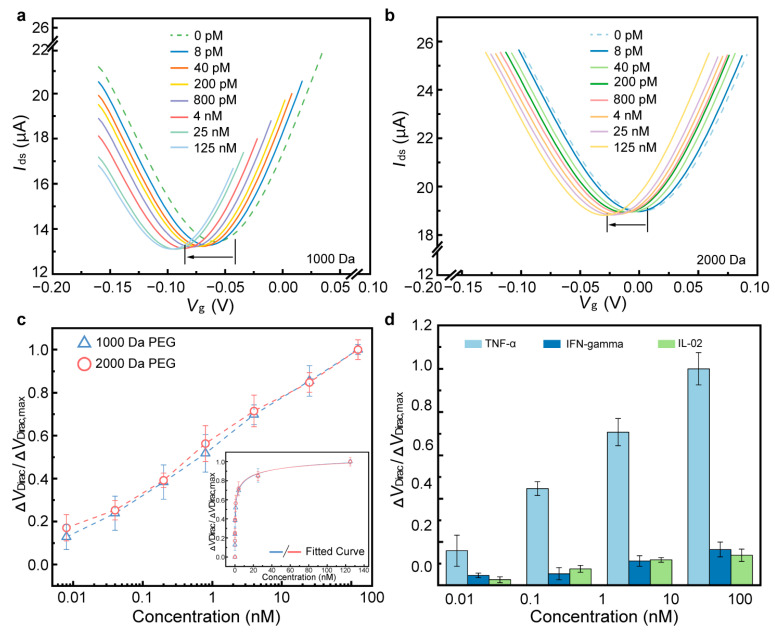
Biomarker detection in PBS. Transfer characteristic curves of the sensing response to different TNF-α concentrations in PBS using the biosensor modified with 1000 (**a**) and 2000 Da PEG (**b**). (**c**) ΔV_Dirac_/ΔV_Dirac,max_ as a function of TNF-α concentrations in PBS. The inset shows the fitted curves of the sensing responses. (**d**) ΔV_Dirac_/ΔV_Dirac,max_ show the responses to different TNF-α concentrations and the control proteins (IFN-γ and IL-002) in PBS.

The detection capability of the biosensor was first investigated in PBS using the biosensor modified with 1000 Da and 2000 Da PEG. TNF-α, a cytokine produced by white blood cells, plays a key role in regulating immune and inflammatory responses [33,34,35]. Studies indicate that abnormal concentrations of TNF-α suggest its critical role in the molecular pathogenesis of cervical carcinoma and offer a potential biomarker for the early diagnosis and therapy of the disease [36,37,38]. The sensing mechanism relies on the structural transformation of the aptamer upon binding with proteins (Figure 3). This guanine-rich oligonucleotide sequence can adopt a compact and stable G-quadruplex structure, formed by two guanine tetrads originating from its loop region. Hence, TNF-α protein (isoelectric points is arounds 6.0), negatively charged in 1 × PBS, closely approached the graphene through binding with the aptamer. This interaction altered the conductivity of the graphene, thereby inducing n-type doping in graphene and resulting in a negative shift in the transfer characteristic curve. As shown in Figure 4a,b, the transfer characteristic curves continuously shifted towards the negative side of the X-axis as the concentration of TNF-α increased from 0.008 to 125 nM. For the biosensor modified with 1000 Da PEG, ΔV_Dirac_ shifted from −58 to −97 mV, and the value of ΔV_Dirac_ decreased from −1 to −31 mV for the biosensor modified with 2000 Da PEG. 

To investigate the binding affinity between the aptamer and the TNF-α protein, a Hill–Langmuir binary binding model was applied to the characterized dissociation constant K_D_ of the biosensor. The non-linear approximation of the fitted curve was calculated based on the Hill–Langmuir equation, which is widely used in biochemical characterization (Figure 4c inset) [39,40].

(1) $$\Delta V_{\mathrm{Dirac}}=\frac{\Delta V_{\mathrm{Dirac},\max}C_{TNF-\alpha }}{K_{D}+C_{TNF-\alpha }}$$

where Δ*V*_Dirac_,_max_ is the saturated shift of *V*_Dirac_, and *C_TNF-α_* is the TNF-α concentration. *K*_D_ represents the affinity between the aptamer and the TNF-α protein; a lower K_D_ value corresponds to a higher binding affinity. The impact of device variations on responses was mitigated by normalizing the Dirac point shift, defined as Δ*V*_Dirac_/Δ*V*_Dirac,max_, which was plotted as a function of TNF-α concentration for the biosensor with PEG modification (Figure 4c) or without PEG modification (Figure S1). From fitted curves, *K*_D_ values were calculated as 1.52 ± 0.35 nM, 1.21 ± 0.44 nM, and 2.82 ± 0.19 nM for biosensors modified with 1000 and 2000 Da PEG, and those without PEG modification in PBS, respectively. The increased value in *K*_D_ for the biosensor without PEG modification suggests that the presence of PEG on the surface contributes to the sensing response due to the increased Debye length [31,32]. Thus, PEG-modified biosensors exhibit a highly sensitive response in biomarker detection, with a lower LOD and response time than seen in most existing methods (Table 1).

Before testing the target protein, it is crucial to validate the biosensor’s specificity and ensure that the sensing signal is generated by targets rather than nontarget molecules. Therefore, control proteins, such as IFN-γ and IL-02, which belong to the same cytokine family as TNF-α, were selected. TNF-α and control proteins were prepared in PBS with concentrations of 0.01, 0.1, 1, 10, and 100 nM, respectively. As shown in Figure 4d, the sensing responses of control proteins were negligible compared with TNF-α. The value of ΔV_Dirac_/ΔV_Dirac,max_ for control proteins was more than six times lower than that for TNF-α, indicating that the biosensor exhibits high specificity for the target biomarker.

### 3.2. Detection of TNF-α in Undiluted Artificial Sweat

**Figure 5:**
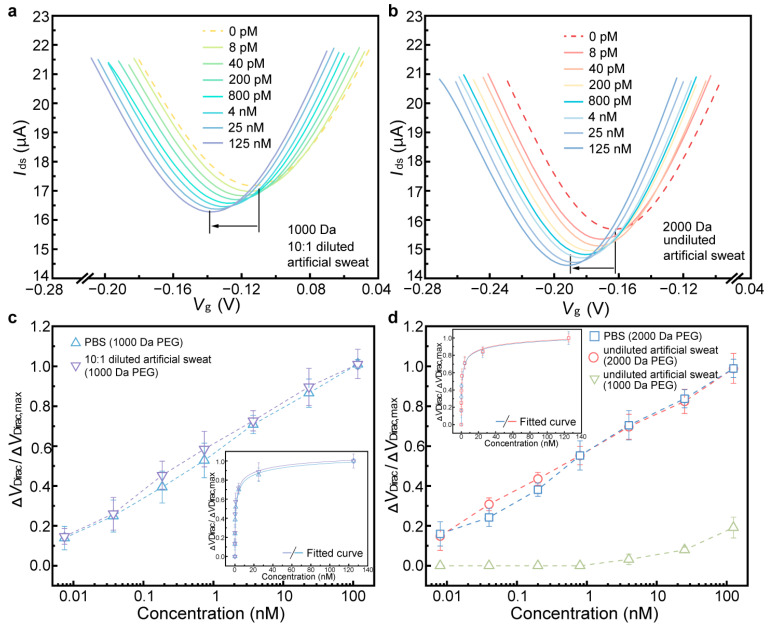
Biomarker detection in artificial sweat. Transfer characteristic curves of sensing responses to different TNF-α concentrations modified with 1000 Da PEG in 10× times diluted artificial sweat (**a**) and modified with 2000 Da PEG in undiluted artificial sweat (**b**). (**c**) ΔV_Dirac_/ΔV_Dirac,max_ as a function of TNF-α concentrations using the biosensor modified with 1000 and 2000 Da PEG in 10× times diluted artificial sweat (**c**) and undiluted artificial sweat (**d**), respectively. The inset shows fitted curves of sensing responses.

To further verify the detection capability of PEG-modified biosensors in the physiological media, we conducted biomarker detection in artificial sweat. The biosensor modified with 1000 Da PEG only offered the capability of biomarker detection in 10 × diluted artificial sweat (Figure 5a), and ΔV_Dirac_ decreased from −114 to −140 mV, which is highly consistent with that tested in PBS (Figure 4a). Based on the fitted curve, K_D_ was calculated to be 1.39 ± 0.12 nM (Figure 5c), differing from the value seen in PBS by 8.6%, which suggested that the detection performance of the biosensor maintains high consistency with the corresponding value tested in PBS. However, the biosensor modified with 1000 Da PEG exhibits a minimal response to TNF-α concentrations below 25 nM in undiluted artificial sweat (Figure 5d), due to it not suppressing nonspecific adsorption. Moreover, the biosensor modified with 2000 Da PEG was tested in the measurement of TNF-α in undiluted artificial sweat (Figure 5b). The shift of ΔV_Dirac_ was 30 mV, the deviation of which, from the maximum ΔV_Dirac_ in PBS (32 mV), was less than 6.3%. K_D_ was estimated to be 1.24 ± 0.39 nM (Figure 5d), differing from the value found in the PBS test (1.21 ± 0.44 nM) by only 2.5% or less. In addition, experiments on biosensors without PEG modification were performed in undiluted artificial sweat (Figure S2). GFET shows the negligible sensing response compared with the biosensor modified with 2000 Da PEG, suggesting that PEG modification successfully suppresses nonspecific adsorption and enables biomarker detection in undiluted physiological media.

The function of larger molecule weights in PEG was also investigated. The value of ΔV_Dirac_ changed from −46 to −77 mV for the 5000 Da PEG modified biosensor, whose deviation from the maximum ΔV_Dirac_ for 2000 Da PEG was less than 3.3% (Figure S3). Based on the fitted curve, K_D_ was estimated to be 1.19 ± 0.23 nM, differing from the value of the 2000 Da PEG-modified biosensor by 4.0% (Figure S2). These results suggest that the 2000 Da PEG would be enough to occupy the vacant area of the graphene surface, thereby enabling a consistent response with the biosensor modified with 5000 or even larger Da PEG. The results above showed that the GFET biosensor maintains high consistency and sensitivity for biomarker detection in undiluted physiological media. This highly desirable behavior for the biosensor could be explained by attributes of the PEG polymer. PEG polymers enable a substantial decrease in the dielectric constant of aqueous solutions, and thus serve to increase the effective Debye screening length for high-ionic-strength solutions [31,32]. Also, PEG formed a permeable layer on the graphene surface, which minimizes nonspecific binding in physiological media by preventing nontarget molecules from attaching to the graphene surface.

### 3.3. Detection of IL-6 in Undiluted Lavage Fluid

**Figure 6:**
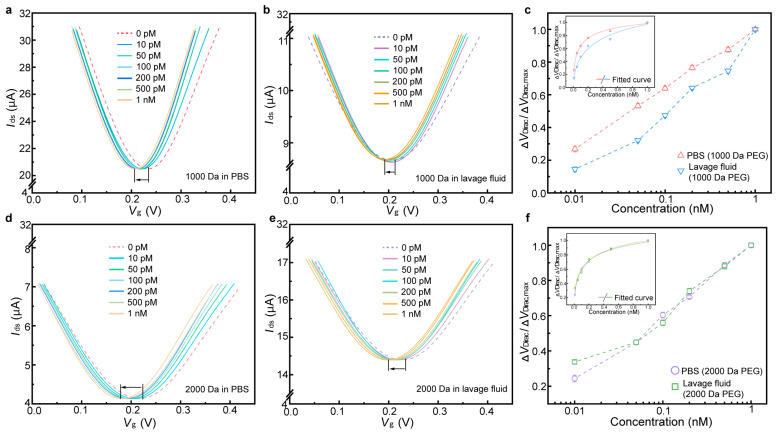
Sensing response of the biosensor to the IL-6 biomarker. Transfer characteristic curves of the biosensor modified with 1000 Da PEG in response to different IL-6 concentrations in PBS (**a**) and undiluted perfusion media (**b**). (**c**) Δ*V*_Dirac_/Δ*V*_Dirac,max_ as a function of IL-6 concentrations in PBS and undiluted perfusion media (1000 Da PEG). The inset shows fitted curves of sensing responses. (**d**) Transfer characteristic curves of the biosensor modified with 2000 Da PEG in response to different IL-6 concentrations in PBS (**d**) and undiluted perfusion media (**e**). (**f**) Δ*V*_Dirac_/Δ*V*_Dirac,max_ as a function of IL-6 concentrations in PBS and undiluted perfusion media (2000 Da PEG). The inset shows fitted curves of sensing responses.

Perfusion medium refers to the covering fluid collected after tissue lavage, which contains various biotargets such as tumor cells, cytokines, and microenvironment components. Detection of such media (e.g., through biochemical testing and immunoassays) offers significant value for the diagnosis, observation, and prognosis of cervical carcinoma [46,47,48]. Particularly, IL-6 (with isoelectric points around 5.0), a representative cytokine, has the capacity to indicate the progression and clinical characteristics of cervical carcinoma [49,50]. Monitoring of IL-6 in PBS was first investigated using the biosensor modified with 1000 and 2000 Da PEG, respectively (Figure 6a and Figure 6d). As the IL-6 concentration increased from 0.1 to 1 nM, the transfer characteristic curves continuously shift towards the negative side of X axis, and the maximum Δ*V*_Dirac,max_ shifts measured were, respectively, 27 (1000 Da PEG modification) and 33 mV (2000 Da PEG modification). Based on the fitted curve, *K*_D_ was calculated to be 84 ± 23 and 188 ± 52 pM in PBS (Figure 6c and Figure 6f), respectively, which shows improvements over the values reported in the previous literature [51,52].

The biosensor was next modified to detect IL-6 in undiluted lavage fluid. For the biosensor modified with 1000 Da PEG (Figure 6b), we observed that the sensing response was shifted by 20 mV, varying from the maximum shift tested in PBS by about 25%. *K*_D_ was calculated to be 570 ± 96 pM, showing a significant difference from the value in PBS (Figure 6c). These results also illustrate that the biosensor modified with 1000 PEG was difficult to completely suppress in terms of nonspecific adsorption, thereby reducing the sensing response in undiluted physiological media. The response of the biosensor modified with 2000 Da PEG holds high consistency with that tested in PBS (Figure 6e). Δ*V*_Dirac_ was shifted by 29 mV, with a variation of less than 12%. The dissociation constant *K*_D_ was estimated to be 194 ± 23 pM in undiluted lavage fluid (Figure 6f), which deviated by less than 3.2% from the *K*_D_ in PBS. These results suggest that the biosensor modified with PEG was able to greatly reduce nonspecific adsorption on the graphene surface. Additionally, it illustrates that the molecular length of the PEG plays a significant role in the sensing response, as the biosensor modified with 1000 Da PEG showed obvious signal attenuation in undiluted lavage fluid compared with 2000 Da PEG modification.

## 4. Discussion

The results above, which show the PEG-modified biosensor’s success or failure in detecting biomarkers in undiluted physiological media, indicate that PEG length (molecular weight) offers a significant impact on biomarker detection rates in the high-ionic-strength solution. These highly desirable biosensor behaviors could mainly be explained as being effects of the suppression of nonspecific adsorption by the PEG polymer. For example, the biosensor modified with 1000/2000 Da PEG maintains a consistent sensing response in high-ionic solution (1 × PBS) (Figure 4). However, the biosensor modified with 1000 Da PEG shows a negligible sensing response for TNF-α concentrations below 25 nM in undiluted artificial sweat, and its sensing response declines more than 20% for IL-6 in undiluted lavage fluid (Figure 5d and Figure 6c). These results illustrate that while Debye length may contribute to sensor response, its role in enabling measurement in physiological media may be secondary compared its role in the suppression of nonspecific adsorption. Longer PEG polymers cover more sites on the graphene surface, thereby enhancing their ability to prevent nonspecific binding. Consequently, the biosensor exhibits heightened sensitivity and specificity in physiological media, suggesting that optimal PEG modification could enable monitoring of various biomarkers in physiological media for point-of-care applications.

## 5. Conclusions

In summary, we demonstrated a general strategy for label-free, sensitive, and specific detection of biomarkers in high-ionic-strength physiological media using a graphene-based field-effect transistor biosensor modified with PEG. The aptamer specifically binds with the biomarker, and induced a change in the carrier concentration of the graphene, which was measured to determine the biomarker concentration. A porous and biomolecule-permeable PEG layer formed on the graphene surface reduced the nonspecific adsorption in physiological media (e.g., undiluted artificial sweat and lavage fluid). The biosensor thus possesses highly sensitive and specific sensing responses to the biomarker in undiluted artificial sweat. In addition, the effect of PEG length on biomarker detection was studied. From the comparison of results, we believe that PEG length is one of the major factors that affects the biosensor’s sensing capability in physiological media. In experiments, the biosensor modified with the aptamer and different lengths of PEG was capable of consistently and sensitively detecting the TNF-α protein in undiluted physiological media, achieving a repeatable LOD as low as 0.13 pM for TNF-a and 0.20 pM for IL-6. These results demonstrate that this strategy is a critical step toward the general application of the graphene-based biosensor in the monitoring of disease biomarkers.

## Figures and Tables

**Table 1:** Comparison of the PEG-modified biosensors with existing methods.

Methods	Materials	Test Time	Limit of Detection	Ref.
Biolayer interferometry-based	Antibody Aptamer	11.5 min	62.5 pM	[41]
Optical sensor	Aptamer	N/A	98.23 pM	[42]
Optical sensor	Aptamer	5 min	2.24 pM	[43]
Electrochemical aptasensor	Aptamer	30 min	315.48 pM	[44]
Microfluidic device	Aptamer	30 min	288.9 pM	[45]
Field-effect transistor	Aptamer	5 min	0.13 pM	This work

## Data Availability

All data are contained within the article.

## Supplementary Materials

The following supporting information can be downloaded at: https://www.mdpi.com/article/10.3390/bios15030138/s1, Figure S1: Δ*V*_Dirac_/Δ*V*_Dirac,max_ as a function of TNF-α concentrations using the biosensor modified without/with 1000 Da and 2000 Da PEG in 1 × PBS; Figure S2: Δ*V*_Dirac_/Δ*V*_Dirac,max_ as a function of TNF-α concentrations using the biosensor modified without/with 1000 Da, 2000 Da and 5000 Da PEG in undiluted artificial sweat. Figure S3: Transfer characteristic curves of the sensing response in undiluted artificial sweat using the biosensor modified with 5000 Da PEG.

## Abbreviations

The following abbreviations are used in this manuscript:

PEG	Polyethylene Glycol
GFET	Graphene-based Field-Effect Transistor
TNF-α	Tumor Necrosis Factor Alpha
IL-6	Interleukin-6
PASE	1-Pyrenebutyric Acid N-hydroxysuccinimide Ester
EDC•HCl	1-Ethyl-3-(3-dimethylaminepropyl) Carbodiimide Hydrochloride
NHS	N-Hydroxysulfosuccinimide
PBS	Phosphate-Buffered Saline
