# Supplementary material for: Aptamer-Based Graphene Field-Effect Transistor Biosensor for Cytokine Detection in Undiluted Physiological Media for Cervical Carcinoma Diagnosis
Source: Biosensors (Basel). 2025 Feb 23;15(3):138. doi: 10.3390/bios15030138 (PMC11939848; doi:10.3390/bios15030138)
Supplement: Supplementary file 1 [file biosensors-15-00138-s001.zip › biosensors-3465702-supplementary.pdf]

Aptamer-Based Graphene Field-Effect Transistor Biosensor for Cytokine Detection in Undiluted Physiological Media for Cervical Carcinoma Diagnosis

Ziran Wang [1,*], Wenting Dai [2], Zaiyu Zhang [1] and Haipeng Wang [1]

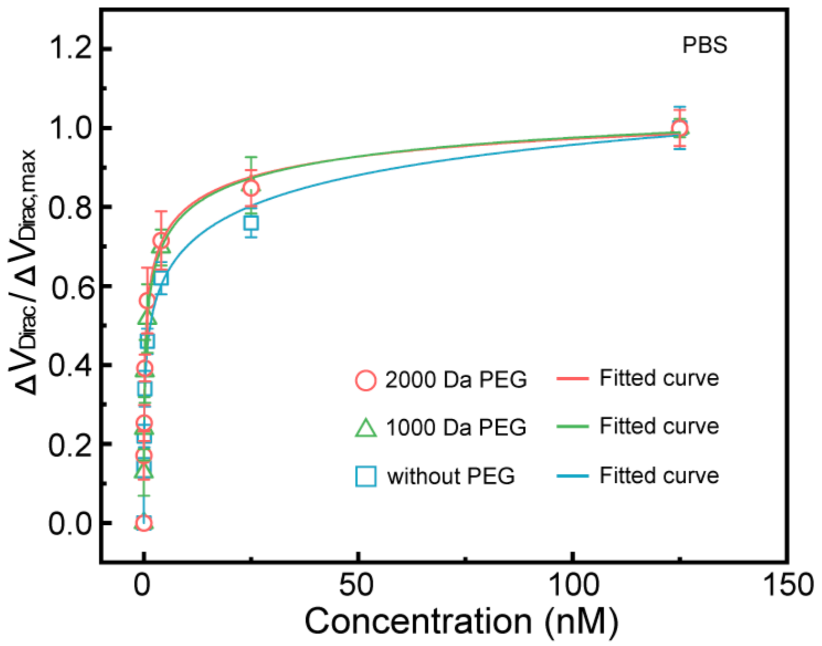

Figure S1. $\Delta V_{Dirac}/\Delta V_{Dirac,max}$ as a function of TNF-α concentrations using the biosensor modified without/with 1000 Da and 2000 Da PEG in 1×PBS.

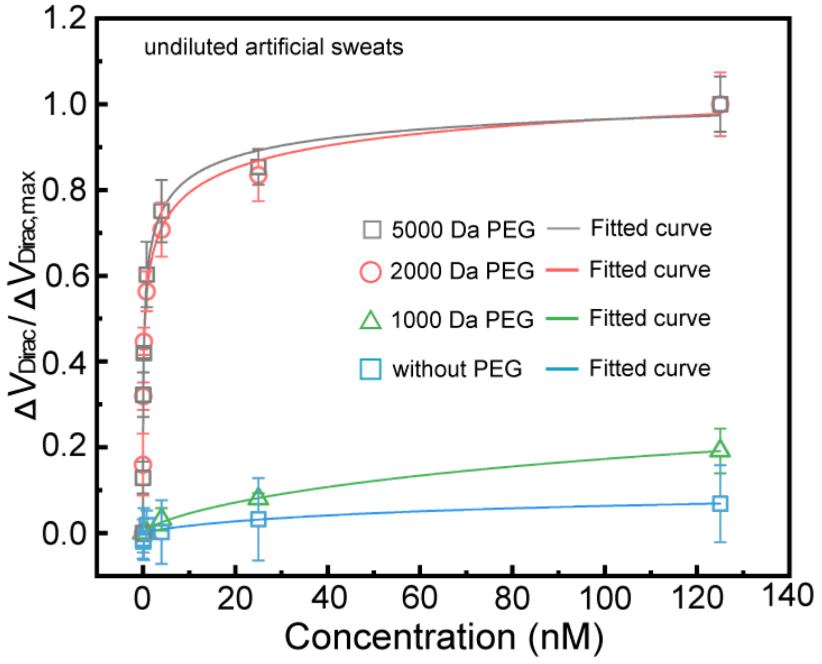

Figure S2. $\Delta V_{Dirac}/\Delta V_{Dirac,max}$ as a function of TNF-α concentrations using the biosensor modified without/with 1000 Da, 2000 Da and 5000 Da PEG in undiluted artificial sweat.

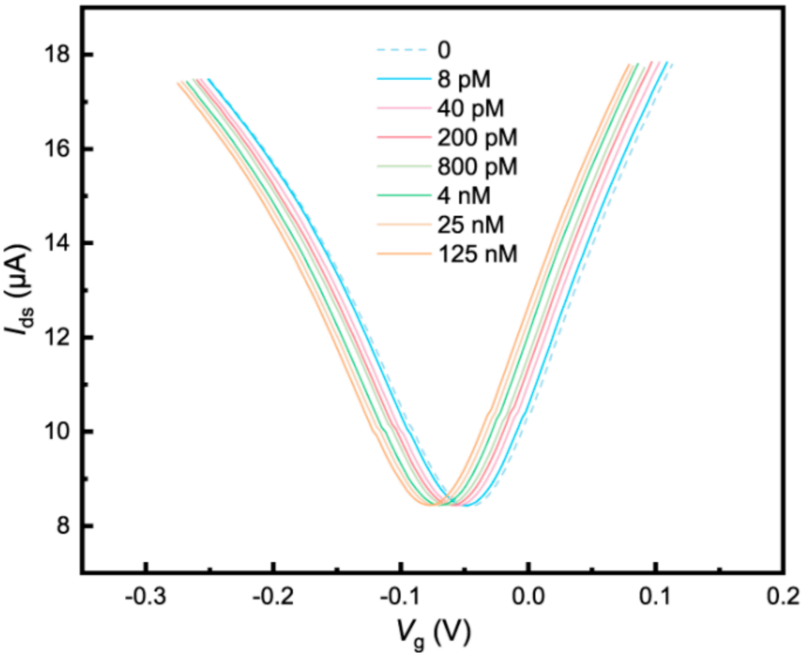

Figure S3. Transfer characteristic curves of the sensing response in undiluted artificial sweat using the biosensor modified with 5000 Da PEG.
